# *Bartonella henselae* Endocarditis in Laos – ‘The Unsought Will Go Undetected’

**DOI:** 10.1371/journal.pntd.0003385

**Published:** 2014-12-11

**Authors:** Sayaphet Rattanavong, Pierre-Edouard Fournier, Vang Chu, Khamthavy Frichitthavong, Pany Kesone, Mayfong Mayxay, Mariana Mirabel, Paul N. Newton

**Affiliations:** 1 Lao-Oxford-Mahosot Hospital-Wellcome Trust Research Unit, Microbiology Laboratory, Mahosot Hospital, Vientiane, Lao People's Democratic Republic; 2 URMITE, IHU Mediterranee-Infection, Faculté de Médecine, Aix-Marseille Université, Marseille, France; 3 Department of Cardiology, Mahosot Hospital, Vientiane, Lao People's Democratic Republic; 4 Faculty of Postgraduate Studies, University of Health Sciences, Vientiane, Lao People's Democratic Republic; 5 Centre for Tropical Medicine and Global Health, Churchill Hospital, University of Oxford, United Kingdom; 6 INSERM U970, Paris Cardiovascular Research Center ­ PARCC, Paris, France; 7 Université Paris Descartes, Sorbonne Paris Cité, Paris, France; 8 Assistance Publique-Hôpitaux de Paris, Hôpital Européen Georges Pompidou, Paris, France; University of California San Diego School of Medicine, United States of America

## Abstract

**Background:**

Both endocarditis and *Bartonella* infections are neglected public health problems, especially in rural Asia. *Bartonella* endocarditis has been described from wealthier countries in Asia, Japan, Korea, Thailand and India but there are no reports from poorer countries, such as the Lao PDR (Laos), probably because people have neglected to look.

**Methodology/Principal Findings:**

We conducted a retrospective (2006–2012), and subsequent prospective study (2012–2013), at Mahosot Hospital, Vientiane, Laos, through liaison between the microbiology laboratory and the wards. Patients aged >1 year admitted with definite or possible endocarditis according to modified Duke criteria were included. In view of the strong suspicion of infective endocarditis, acute and convalescent sera from 30 patients with culture negative endocarditis were tested for antibodies to *Brucella melitensis,* *Mycoplasma pneumoniae,* *Bartonella quintana,* *B. henselae,* *Coxiella burnetii* and *Legionella pneumophila*. Western blot analysis using *Bartonella* species antigens enabled us to describe the first two Lao patients with known Bartonella henselae endocarditis.

**Conclusions/Significance:**

We argue that it is likely that *Bartonella* endocarditis is neglected and more widespread than appreciated, as there are few laboratories in Asia able to make the diagnosis. Considering the high prevalence of rheumatic heart disease in Asia, there is remarkably little evidence on the bacterial etiology of endocarditis. Most evidence is derived from wealthy countries and investigation of the aetiology and optimal management of endocarditis in low income countries has been neglected. Interest in *Bartonella* as neglected pathogens is emerging, and improved methods for the rapid diagnosis of *Bartonella* endocarditis are needed, as it is likely that proven *Bartonella* endocarditis can be treated with simpler and less expensive regimens than “conventional” endocarditis and multicenter trials to optimize treatment are required. More understanding is needed on the risk factors for *Bartonella* endocarditis and the importance of vectors and vector control.

## Author Summary

Infection of heart valves (endocarditis) with bacteria is an important condition, especially afflicting those with rheumatic heart disease, and has a high mortality if untreated. Most of the evidence for optimal antibiotic and surgical management comes from wealthy countries. There are no published data from poorer countries in SE Asia despite a high burden of rheumatic heart disease. We investigated the bacterial infections of heart valves in the Lao PDR (Laos) through heart ultrasound scans and analysis of patients' blood. We provide evidence of infection with the poorly understood bacteria *Bartonella henselae* (the cause of cat scratch disease) in two patients from Laos. We argue that it is likely that *Bartonella* endocarditis is more widespread than appreciated, as there are few laboratories in Asia able to make the diagnosis. This is important as it is likely that proven *Bartonella* endocarditis can be treated with simpler and less expensive regimens than “conventional” endocarditis. There have been great advances in the wealthy world in the diagnosis and treatment of endocarditis but these have not been assessed or implemented in poorer countries. More evidence on the causes and optimal management of endocarditis in low income countries is needed.

## Introduction

There has been emerging interest in the importance of *Bartonella henselae* endocarditis in the wealthier countries of Asia [1]–[3] but few data from poorer countries. We describe two patients admitted with this condition at Mahosot Hospital, Vientiane, Lao PDR (Laos), and discuss the public health implications.

## Methods

As part of a blood culture and infectious disease liaison service at Mahosot Hospital we identified thirty patients with culture negative endocarditis 2006–2012 [4]. Mahosot Hospital (17.960 N, 102.612 E) is a primary-tertiary care teaching hospital of ∼400 beds including cardiology and infectious disease wards. Patients were identified through liaison between the microbiology laboratory and the wards, especially with those performing cardiac ultrasound. Those aged >1 year admitted to Mahosot Hospital with definite or possible endocarditis according to modified Duke criteria were included in a retrospective study 2006–2012 and, since then, in a prospective study. The hospital has trans-thoracic echocardiography, with occasional trans-oesophageal echocardiography. Blood cultures were performed as described [5]. The clinical significance of positive blood cultures was determined by physicians at the time of the result. Acute and convalescent (when available) sera from those with culture negative endocarditis were tested for antibodies to *Brucella melitensis*, *Mycoplasma pneumoniae*, *Bartonella quintana*, *B. henselae*, *Coxiella burnetii* and *Legionella pneumophila* were tested for by indirect immunofluorescence assay (IFA) [6]. Sera exhibiting phase 1 immunoglobulin G (IgG) titers >1∶800 for *C. burnetii* or IgG titers >1∶800 for *Bartonella quintana* and *B. henselae* were considered as highly predictive of endocarditis caused by these microrganisms. We also considered total antibody titers≥1∶256 for *L. pneumophila* as positive. Specific antibodies to *Brucella melitensis* and *Mycoplasma pneumoniae* were detected with an immunoenzymatic antibody test and the Platellia *M. pneumoniae* IgM kit (Bio-Rad, Marnes-la-Coquette, France), respectively. Titers of >1∶200 were considered positive. When the results of the tests described above were negative, or when IgG titers to *Bartonella* were ≤1∶800, we performed Western blot using *B. henselae* and *B. quintana* antigens, followed by cross-adsorption as described [6]. Patients with *Bartonella* endocarditis exhibit specific Western blot profiles, different from other types of *Bartonella* infections [7].

### Ethics Statement

Patients gave informed written consent for a prospective description of the causes of infection approved by the Oxford Tropical Research Ethics Committee, UK, and the National Ethics Committee for Health Research, Laos.

## Results

Of the 30 patients tested, two were Western Blot positive for *B. henselae*. They had definite and possible endocarditis, respectively, by modified Duke criteria.

**Figure 1:**
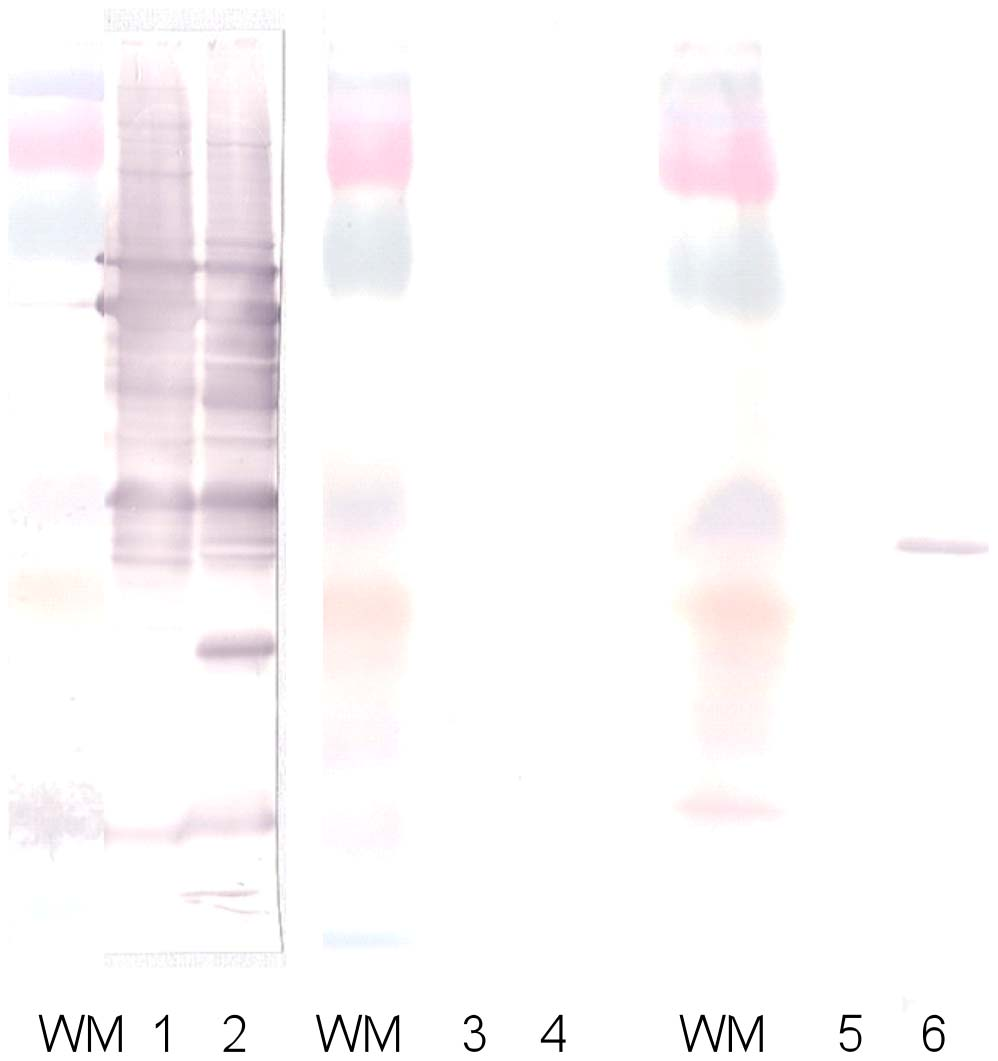
Western blot performed from the first patient's serum before and after cross-adsorption. WM = Weight marker; lanes 1, 3 and 5: *B. quintana* antigen; lanes 2, 4 and 6: *B. henselae* antigen; lanes 1 and 2: unadsorbed serum; lanes 3 and 4: serum adsorbed with *B. henselae*; lanes 5 and 6: serum adsorbed with *B. quintana*.

In 2012 a previously healthy 57-year-old army officer from Pakse, southern Laos, was admitted with one month of fever, headache, myalgia, back pain, productive cough and 4 days of chills and dyspnea. On examination he was afebrile, normotensive but with a pansystolic (3/6) murmur at the mitral and tricuspid areas with clear lungs and no peripheral signs of endocarditis. His admission peripheral blood count was white blood count (WBC) 7.2×10^9^/L, haemoglobin 7.4 g/dL, mean cell volume 79 fL, mean cell haemoglobin 24.6 pg and platelets 159×10^9^/L. Transthoracic echocardiogram showed a vegetation on the mitral valve (maximum length 1.9 cm), with mild mitral regurgitation, mild aortic and tricuspid valve regurgitation. Three sets of blood cultures incubated for 7 days showed no growth. The patient exhibited IgG titers of 1∶400 to both *B. henselae* and *B. quintana* in acute serum, and then 1∶200 in the convalescent serum. He also had a specific Western blot profile for *B. henselae* endocarditis (Fig. 1). He was treated with intravenous ceftriaxone 2 g once a day for 6 weeks and gentamicin 240 mg/d for 2 weeks and was well at one year follow up.

A 69-year old housewife from Xaysettha district, Vientiane Capital, was admitted in 2008 with two months of fever, headache, arthralgia, back pain, myalgia, jaundice, diarrhea, productive cough and dyspnea, with a history of hypertension. She had been treated with ceftriaxone before admission. On examination she was afebrile and normotensive but with a systolic heart murmur. Her admission peripheral blood count showed hematocrit 32%, WBC 4.5×10^9^/L (lymphocytes 62%) and platelets 85×10^9^/L. Trans-thoracic echocardiogram demonstrated significant mitral and aortic valve disease but no vegetation: thickening of mitral valve (D∼5–6 mm) with moderate to severe mitral regurgitation (regurgitant volume = 57 ml/s) and thickening of aortic valve with aortic regurgitation grade 1–2/4, and elevated estimated pulmonary artery systolic pressure of 70 mmHg. Five pairs of blood cultures, incubated for 7 days, showed no growth. The patient had negative IFA results to *Bartonella* species but her Western blot profile was typical of *B. henselae* endocarditis. She was treated with ceftriaxone 2 g/day for 14 days with resolution of symptoms but died of gastric perforation in 2011.

## Discussion

In Asia, *Bartonella* endocarditis has been described from Japan (*B. henselae* [1]), Korea (*B. quintana* [2]), Thailand (*B. henselae* [3]) and India (*B. quintana* [8]), but we found no reports from Cambodia, Lao PDR, Vietnam, Burma/Myanmar, China, Indonesia, Taiwan or Malaysia (Pubmed using these country names and ‘*Bartonella*’ ‘endocarditis’). Two additional *Bartonella* species, resembling those reported as causing endocarditis elsewhere, have been described from Thailand, *B. vinsonii* from humans and *B. elizabethae* from rodents [9]. *B. henselae* occurs in cat fleas, and possibly ticks [10], and this pathogen has been recorded in fleas from Malaysia and Thailand but not elsewhere in mainland Asia [11], [12].

It is likely that *Bartonella* endocarditis is more widespread than appreciated as few have looked and laboratories in Asia able to make the diagnosis are infrequent. The diagnosis of culture-negative endocarditis is most effectively performed from valve tissue, but there are few cardiac surgeons in rural Asia. Considering the high prevalence of rheumatic heart disease in Asia there is remarkably little evidence on the bacterial etiology of endocarditis. Prospective investigations, especially at centers able to perform valvular surgery, would inform prevention and treatment policies for these very large populations devoid of evidence. Improved methods for the rapid diagnosis of *Bartonella* endocarditis are needed, as it is likely that proven *Bartonella* endocarditis can be treated with simpler and less expensive regimens (e.g. gentamicin for 2 weeks plus oral doxycycline for 6 weeks) than ‘conventional’ endocarditis [13], but multicenter trials to optimize treatment are also needed.
